# Supplementary material for: Concomitant Inhibition of FASN and SREBP Provides a Promising Therapy for CTCL
Source: Cancers (Basel). 2022 Sep 16;14(18):4491. doi: 10.3390/cancers14184491 (PMC9496997; doi:10.3390/cancers14184491)
Supplement: Supplementary file 1 [file cancers-14-04491-s001.zip › cancers-1841667-supplementary.pdf]

Supplementary figures and tables

Supplementary figures

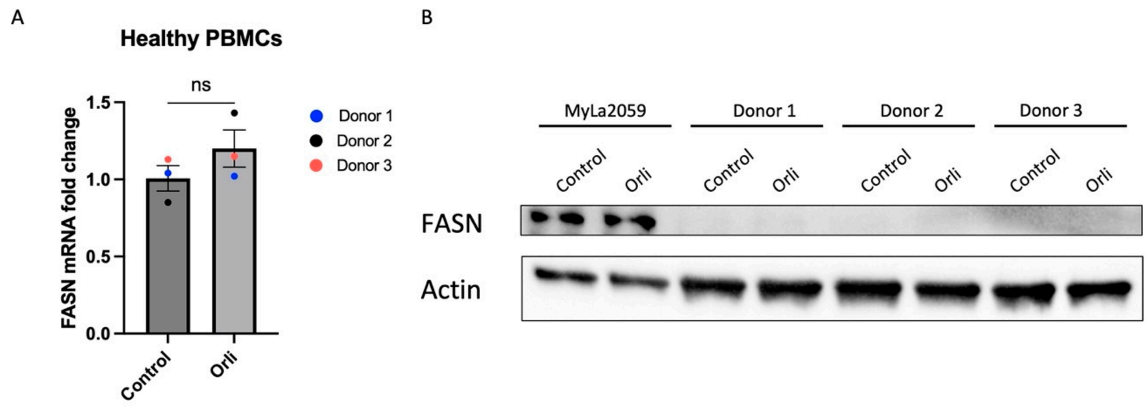

Supplementary Figure S1. Analysis of FASN mRNA (A) and protein expression (B) in PBMCs from healthy donors. CTCL cell line MyLa2059 and healthy PBMCs were incubated with Orlistat (Orli) for 24 hours prior to analysis of mRNA expression of FASN by qPCR, and protein expression by Western blotting, as described in materials and methods. Expression of GADPH was used as housekeeping for analysis of mRNA expression, and actin was used as loading control for analysis of protein expression. Statical significance between healthy PMBCs control or treated with Orlistat (Orli) were tested with two-sided Student's t test.

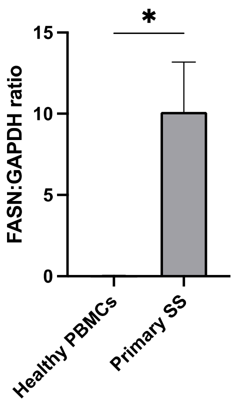

Supplementary Figure S2. FASN expression in PBMCs from healthy donors or primary SS. mRNA was purified from PBMCs from healthy donors and SS patients, and expression of FASN mRNA was determined by qPCR as described in materials and methods. GAPDH was used as housekeeping gene for analysis of mRNA expression. Statical significance between healthy PMBCs and primary SS samples were tested with two-sided Student's t test (*$p \leq 0.05$).

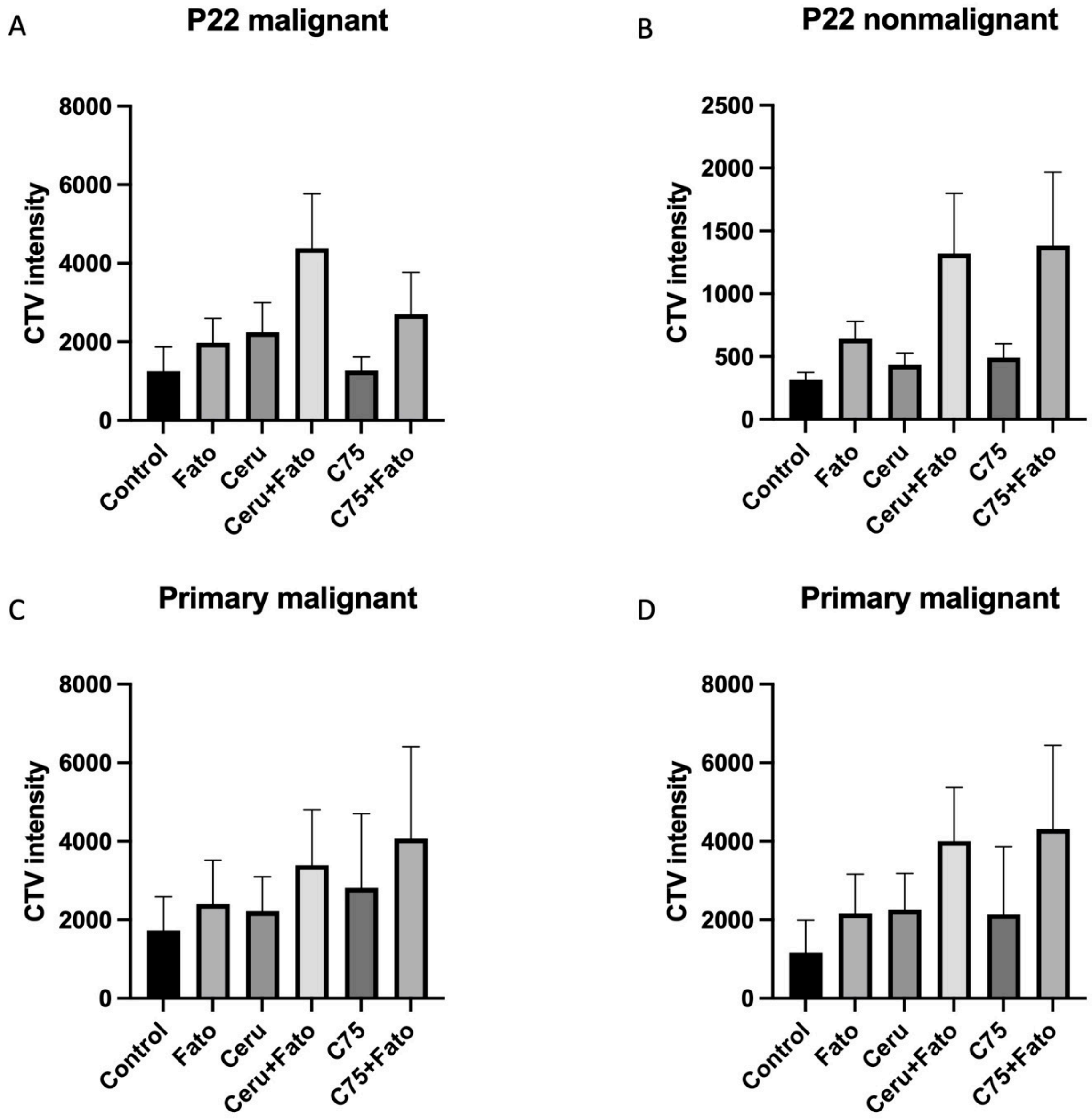

Supplementary Figure S3. Proliferation analysis on primary cells based on CTV raw number. Instead of using CTV fold change normalized by control, this figure directly showed the raw intensity number of CTV which was still like what is shown in Figure 6. Statical significance testing was performed with a one-way ANOVA (Tukey´s multiple compari-son) test described in materials and methods.

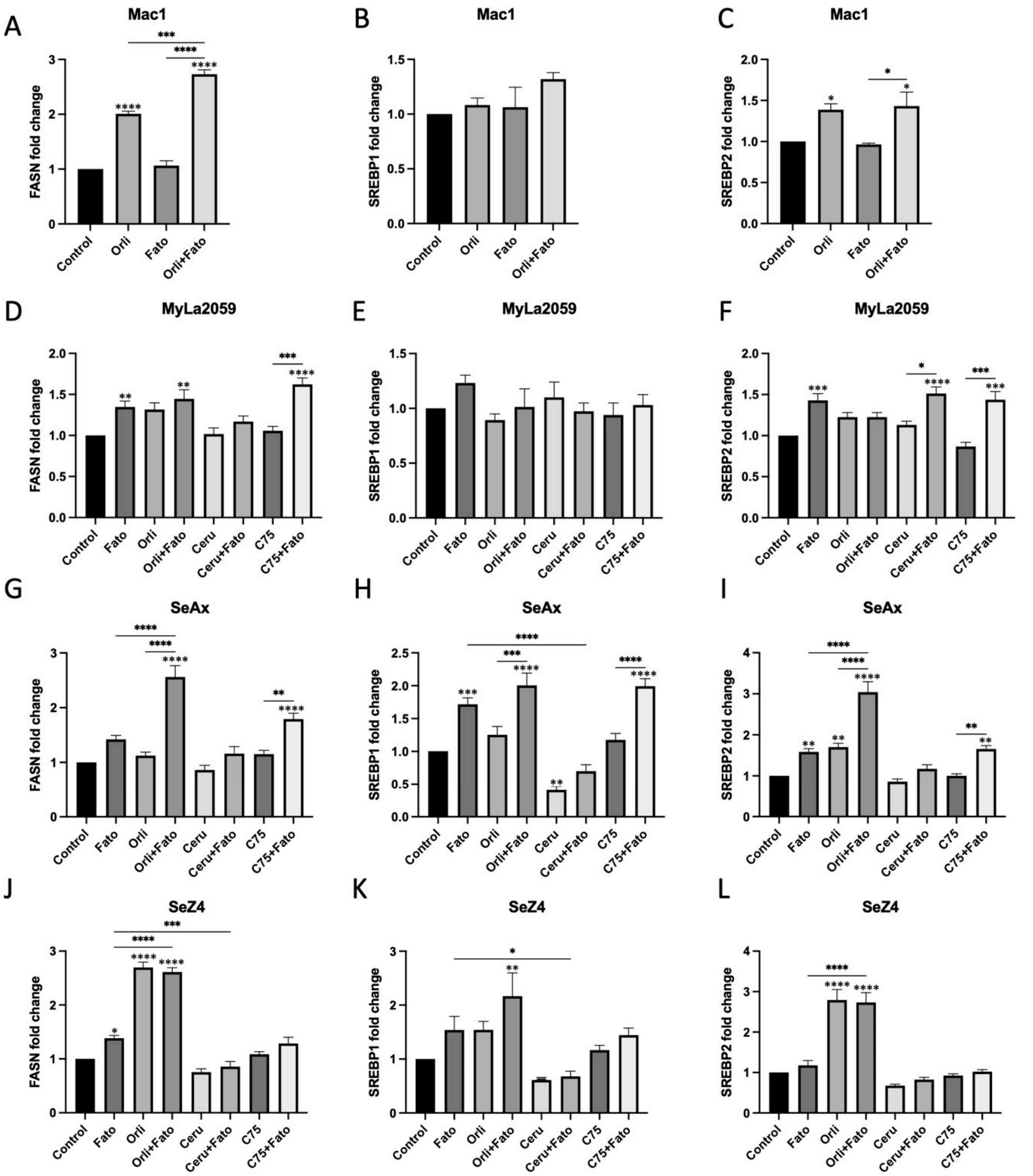

Supplementary Figure S4. FASN, SREBP1 and SREBP2 mRNA expression after treatment of FASN/SREBP inhibitors for 24 hours. Although Cerulenin (Ceru) and C75 did not increase the expression of FASN in every cell line, while Orlistat (Orli) and Fatostatin (Fato) prone to upregulate FASN, the combination of Fatostatin and either Orlistat, Cerulenin or C75 tended to increase the FASN expression. Furthermore, the increase or decrease of FASN was always accompanied with the increase of SREBP1 and/or SREBP2. Statical significance was calculated with a one-way ANOVA test with Tukey´s multiple comparison as described in materials and methods. Asterisks above each column in (B-H) indicates the significance of difference compared to the control.

Bars with asterisks signify the difference between an inhibitor and any combination with this inhibitor (*$p \leq 0.05$; **$p \leq 0.01$; ***$p \leq 0.001$; ****$p \leq 0.0001$).

Supplementary table

	MyLa2059	Mac-1	Mac-2a	PB2B	HH	SeZ4	SeAx	Healthy PBMCs
Orlistat	0.01961	0.0003792	0.01764	0.004616	0.004788	ND	ND	NC
Cerulenin	0.006492	0.005731	0.006193	0.005870	0.006164	0.01164	0.01769	0.007488
C75	0.08425	0.01993	0.02945	0.028440	0.0311	0.05787	0.03046	0.0313

Supplementary Table S1. IC_{50} values of the three FASN inhibitors on the different CTCL cell lines and PBMCs from healthy donor. IC_{50} (M) were determined by non-linear fitted [inhibitor] vs. response curves for variable slope (four parameters) using GraphPad Prism 9 software. IC_{50} for Healthy PBMCs treated with Orlistat could not be calculated (NC). ND, not determined.
